# Unlocking the Viral Universe: Metagenomic Analysis of Bat Samples Using Next-Generation Sequencing

**DOI:** 10.3390/microorganisms11102532

**Published:** 2023-10-10

**Authors:** German V. Roev, Nadezhda I. Borisova, Nadezhda V. Chistyakova, Matvey R. Agletdinov, Vasily G. Akimkin, Kamil Khafizov

**Affiliations:** 1 Central Research Institute of Epidemiology, 111123 Moscow, Russia; 2 Moscow Institute of Physics and Technology, National Research University, 115184 Dolgoprudny, Russia; 3 A.N. Severtsov Institute of Ecology and Evolution, Russian Academy of Sciences, 119071 Moscow, Russia

**Keywords:** viruses, NGS, metagenomics, bioinformatics, bats, SMART

## Abstract

Next-generation sequencing technologies have revolutionized the field of virology by enabling the reading of complete viral genomes, extensive metagenomic studies, and the identification of novel viral pathogens. Although metagenomic sequencing has the advantage of not requiring specific probes or primers, it faces significant challenges in analyzing data and identifying novel viruses. Traditional bioinformatics tools for sequence identification mainly depend on homology-based strategies, which may not allow the detection of a virus significantly different from known variants due to the extensive genetic diversity and rapid evolution of viruses. In this work, we performed metagenomic analysis of bat feces from different Russian cities and identified a wide range of viral pathogens. We then selected sequences with minimal homology to a known picornavirus and used “Switching Mechanism at the 5′ end of RNA Template” technology to obtain a longer genome fragment, allowing for more reliable identification. This study emphasizes the importance of integrating advanced computational methods with experimental strategies for identifying unknown viruses to better understand the viral universe.

## 1. Introduction

The advent of next-generation sequencing (NGS) technologies has not only revolutionized numerous fields of biology and medicine but has also made significant advances in virology. This innovation allows for the exploration of a large number of viruses. As the cost of NGS decreases [1], large-scale metagenomic studies, including those conducted to identify novel viral pathogens, are becoming increasingly feasible. Metagenomic sequencing boasts the advantage of not relying on specific probes or primers for virus detection, thus potentially enabling the discovery of any virus present in a sample, irrespective of its known or unknown status [2]. This unique attribute has positioned metagenomic sequencing as a primary method for detecting both recognized and new viruses [3,4].

Nonetheless, analyzing metagenomic data poses significant hurdles, particularly in the context of detecting new viruses. In addition to the high proportion of host and bacterial genome reads, a significant number of reads in the metagenomic dataset often have no meaningful similarity to genomes in existing databases, making them difficult to identify. This so-called “dark matter” of metagenomics plays a crucial role in virus detection and characterization. Most existing bioinformatic tools for virus identification mainly depend on a homology strategy, that is, they identify viruses based on their genetic similarity to known viruses. However, these tools might fail to detect a virus if it is sufficiently diverse from the previously known ones. This constitutes a significant drawback due to the enormous genetic diversity and rapid evolution of viruses [5]. As a plausible solution to this problem, machine learning techniques are currently being explored [6,7,8,9]. These methodologies facilitate the unveiling of new viruses by considering aspects beyond just genetic sequence similarity. For instance, they have the ability to spot distinctive patterns in how viral genes are organized or in particular motifs of viral proteins, irrespective of the absence of similar matches in current viral genome databases. It is important to realize that machine learning methods have their own nuances and limitations, making it necessary to use them in tandem with other techniques to confirm the existence and signatures of new viruses. Despite these obstacles, the enormous potential of this field is undeniable, and we eagerly anticipate stimulating breakthroughs in the immediate future. Moreover, the amalgamation of sophisticated computational methods and experimental strategies could hold the key for demystifying the viral “dark matter” and broadening our knowledge of the viral universe. For instance, machine learning techniques can be deployed to identify “presumptively viral” reads within metagenomic sequencing data. Once such prospective novel viral sequences have been identified, experimental methodologies can be utilized to procure lengthier fragments of the viral genome sequence, which can subsequently be compared to known viruses for homology. For this objective, techniques centered around DNA/RNA amplification from a single primer followed by NGS, like SISPA (the acronym stands for Sequence-Independent, Single-Primer Amplification), SPIA (single primer isothermal amplification), and SMART (switching mechanism at the 5′ end of the RNA transcript) [10,11,12,13] can be implemented. The latter approach provides the opportunity to use just one RNA-specific primer, and as a result of additional manipulations (see Section 2 for details), to obtain a longer segment of the desired nucleotide sequence. Bioinformatics tools can then be used to analyze the sequence, to compare it with other known virus genomes, and to determine its possible origin and characteristics.

While the identification and characterization of new viruses certainly possess academic value, another key application lies in employing this knowledge in the realms of epidemiology and medicine to anticipate and ward off viral disease outbreaks, as well as to develop diagnostic tests and effective vaccines. Based on recent events, there is heightened focus on studying zoonotic viruses, considering that their transmission rate among humans has significantly escalated. Numerous domestic and wild animals can act as viral infection sources; however, bats have been particularly recognized over the past few decades as significant reservoirs of zoonotic viruses, such as Hendra [14], Nipah [15], SARS [16], MERS [17], Ebola [18], and, in 2019, SARS-CoV-2 [19], which caused the COVID-19 pandemic. Bats have a unique immune system that allows them to tolerate a large number of viruses without clinical signs of disease. This ability is thought to be due to a balance between their defense mechanisms and a special type of immune tolerance [20]. Their immune response, particularly the interferon system, which is a part of the innate immune response, appears to be always “on”, unlike other mammals in which it is “on” only in response to infection. This constant presence of an antiviral state allows bats to control viral replication without the devastating inflammatory response that is often the actual cause of disease in other mammals, including humans [21,22]. Other animals, such as reptiles, insects, birds, rodents, and primates, can also serve as virus reservoirs, but factors such as temperature conditions and behavior often limit these viruses’ potential to spread among humans. However, there are exceptions, such as West Nile virus, that may be transmitted to humans from birds via mosquitoes [23].

In this work, we performed metagenomic analysis of bat feces obtained from different cities of the Russian Federation and identified a large diversity of viruses, including mammalian, plant, and insect viruses. It is important to note that the results are worth considering keeping in mind that fecal samples more often contain “environmental” viral profiles than host-specific pathogenic viruses. Notably, a significant number of reads in the sequencing data have no homology with known genomes of viruses or other organisms, further emphasizing that a significant fraction of viruses remains unexplored. A specific primer was designed from one of the reads with barely detectable homology to a fragment of the picornavirus genome and used in combination with the SMART method [13]. Subsequent sequencing of the SMART product yielded an elongated fragment of the picornavirus genome, allowing more confident identification of the virus. This technique can be extended to many other candidate reads, providing an opportunity to expand our knowledge of the diversity of viruses on the planet.

## 2. Materials and Methods

### 2.1. Sample Collection

Fecal samples were collected from the following species: *Nyctalus noctula* (12 samples from Saratov region and 3 from Rostov), *Vespertilio murinus* (1 sample from Voskresensk, 2 samples from Naro-Fominsk, and 1 sample from Moscow), and *Pipistrellus kuhlii* (1 sample from Astrakhan).

Ethical permission for this research (number 50 dated 7 August 2021) was approved by the Severtsov Institute Bioethics commission, and all procedures were performed accordingly.

### 2.2. Samples Preparation and Metagenomic Sequencing

RNA was isolated from samples using the QIAamp Viral RNA kit (Qiagen, Hilden, Germany). Ten μL of undiluted RNA specimen was used as input for first strand synthesis with the Reverta-L RT reagents kit (AmpliSense, Moscow, Russia). Second strand synthesis was performed using Second Strand Synthesis Module (NEB #E611, Ipswich, MA, USA). M220 Focused-ultrasonicator (Covaris, Woburn, MA, USA) was used to fragment DNA to ~550 bp. Paired-end sequencing libraries were constructed with NEBNext^®^ Ultra™ End Repair/dA-Tailing Module (NEB E7546L, Ipswich, MA, USA), NEBNext^®^ Ultra™ Ligation Module (NEB E7595L, Ipswich, MA, USA), Y-shaped adapters compatible with IDT for Illumina Nextera DNA UD Indexes, and NEBNext^®^ Ultra™ II Q5^®^ Master Mix (NEB M0544X, Ipswich, MA, USA), for barcoding PCR, according to the manufacturer’s instructions. The quality and fragment length distribution of the obtained libraries were evaluated with Agilent Bioanalyzer 2100 (Agilent Technologies, Santa Clara, CA, USA). The first several libraries were sequenced on an Illumina MiSeq instrument using v3 600 cycle reagent kit (Illumina, San Diego, CA, USA). The other libraries were sequenced on an Illumina NextSeq 2000 instrument using P2 300 cycle reagent kit (Illumina, San Diego, CA, USA). Sequencing depth varies among samples and highly depends on the quality of a sample and on the sequencing machine used. Since Illumina NextSeq 2000 generates much more data than Illumina MiSeq, samples sequenced on NextSeq have significantly higher sequencing depths. Final output from NextSeq was 9300 Mb per sample on average (31 million paired-end reads) and from MiSeq 1600 Mb per sample (2,7 millions paired-end reads).

To apply the SMART technology to the samples, we decided to use Mint cDNA synthesis kit (Evrogen JSC, Moscow, Russia) with the replacement of the 3′-primer with another specific one. A contig with a low degree of homology to previously known viruses was selected from the *1_N. noctula_miseq_Saratov* sample using the BLASTX tool with the Mask low-complexity regions option turned off. This contig was therefore classified as Picornaviridae sp. with E-value equal to 0.0001 and amino acid identity of 29.4%. Only four reads out of 3,704,908 were mapped to this contig. One of these reads (Supplementary Material S1) was selected for primer construction (AAGCAGTGGTATCAACGCAGAGTAC). When mapping the read with BLAST to picornavirus genome, it was detected that it mapped in the reverse orientation to the virus. Picornaviruses possess an RNA genome with positive polarity, so a reverse primer was selected for SMART amplification to reverse-complement the sequence of the read. It was then tailed with a Mint adapter to facilitate its amplification and sequencing. The final sequence of the primer with the adapter was AAGCAGTGGTGGTATCAACGCAGAGAGTAC-AGGTTTGACAATGCAGCAGA.

Next, following the selection of a picornavirus-like read in the metagenomic data and primer construction, we applied the SMART method using a complementary primer and the Mint cDNA synthesis kit (Evrogen, Russia) in accordance with the manufacturer’s guidelines. SMART product sequencing libraries were prepared using the Nextera XT kit. The sequencing was performed on the Illumina MiSeq, utilizing the V2 300 cycle reagent kit.

### 2.3. Bioinformatics Analysis of Metagenomic Sequencing Data

**Figure 1:**
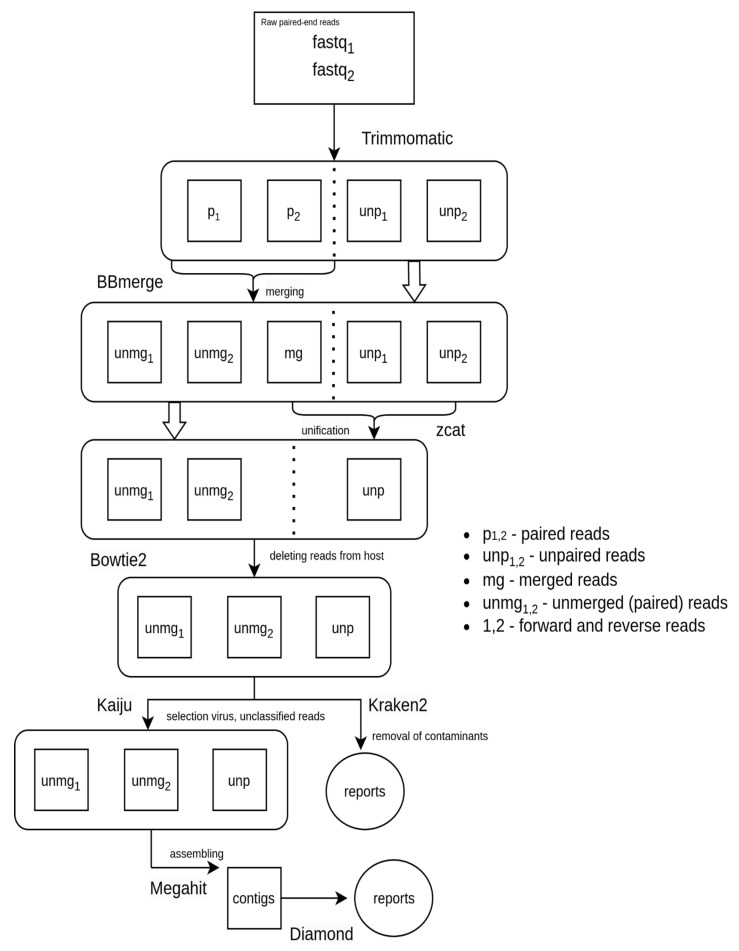
Schematic representation of the pipeline for processing the results of metagenomic sequencing. First, the raw data (fastq1 and fastq2) are trimmed using the Trimmomatic program. *p1* and *p2* are forward and reverse reads that passed quality control, respectively. *unp1* and *unp2* are reads in which only one of the reads passed quality control. BBmerge attempts to combine the paired reads from *p1* and *p2* into a single read. This is only possible for those pairs in which the reads overlap. *mg* are those reads that succeeded in merging and *unmg* are those that failed. The zcat program is then used to merge the files *mg*, *unp1*, and *unp2* into a single file named unp. In the next step, all reads that were mapped to the host genome were removed from the subsequent analysis. Kraken2 was used to taxonomically classify the reads. The Kaiju program was used to obtain only viral and unclassified reads to perform contig assembly. The contigs were then assembled using the Megahit program, after which the Diamond program was applied.

We applied a series of processing steps (Figure 1) to the raw reads using Trimmomatic v0.39 [24], which included adapter trimming with the ILLUMINACLIP option, and the application of LEADING:7, TRAILING:7, SLIDINGWINDOW:4:20, and MINLEN:40 options. Trimmomatic generates four output files for each sample, consisting of two paired-end reads files and two unpaired reads files. We then merged the paired-end reads files using the BBmerge [25] program from the BBtools v38.96 package, applying the maxstrict = t option. The merged reads were subsequently concatenated with the two unpaired reads files from Trimmomatic, resulting in each sample being represented by three files: two unmerged paired-end reads files and one unpaired file.

To perform host reads filtration, reference genomes for *Pipistrellus kuhlii* (GCF_014108245.1) and *Myotis myotis* (GCF_014108235.1) were downloaded from RefSeq (https://www.ncbi.nlm.nih.gov/refseq/, accessed on 18 July 2023). *Myotis myotis* was used because genomes for *Nyctalis noctula* and *Vespertilio murinus* were not available at the time of this study. Fasta files were then concatenated and indexed with bowtie2 v2.4.4 [26]. The subtraction of host reads was executed separately for paired reads, using the –un-conc option, and for unpaired reads, using the—un option, with the assistance of bowtie2.

We employed Kraken2 [27] for taxonomic classification, utilizing the NCBI nt nucleotide database, which was downloaded on the 2nd of March, 2023. We managed paired and unpaired reads separately, employing the –paired option for the paired reads. For each sample output, files for paired and unpaired reads were merged. To exclude possible contaminants (human viruses that we also actively work with in the laboratory such as viruses from *Sarbecovirus* subgenus, *Influenza A* and *B viruses*, and *Human Hepatitis B virus*), reads that were categorized into taxa corresponding to these contaminants were excluded from these files. The final Kraken2 reports were obtained using the make_kreport utility from KrakenTools.

**Figure 2:**
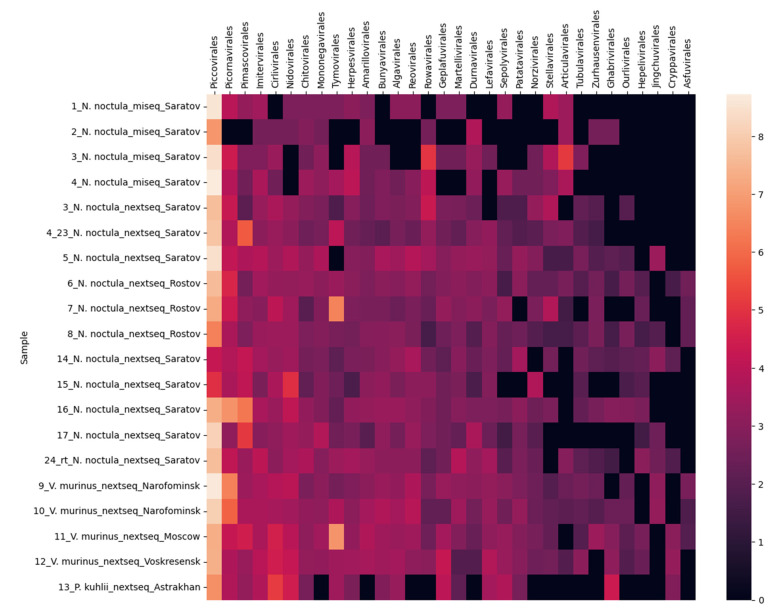
Content of different virus orders in the tested fecal samples. The heatmap shows viral abundance (counted as a percentage of the number of reads from the sample in a particular viral order, multiplied by 10^9^ and displayed as a decimal logarithm). The lighter the color, the higher the viral order content.

We analyzed Kraken2 reports using a custom Python script. In essence, viral taxa at the order level were initially chosen if at least one of the samples had a minimum of 10 hits. Subsequently, nucleotide sequences of phages (*Caudoviricetes, Mindivirales,* and *Petitvirales*), as well as reads corresponding to *Ortervirales*, which encompasses retroviruses, were excluded from these selected taxa. For every sample, we computed the ratio of reads corresponding to each of the aforementioned orders to the total number of reads. These values were then scaled by a factor of 10^9^, followed by the calculation of the decimal logarithm. The derived data were subsequently utilized to construct a heatmap (Figure 2).

To obtain viral contigs, we decided to select only those reads that are identified as viral or unclassified. For this purpose, host-cleaned reads were analyzed by Kaiju v1.9.2 [28] using the nr_euk database (version from 10 May 2023), and only viral and unclassified reads were selected. From these, contigs were assembled using MEGAHIT v1.2.9 [29]. Contigs were classified using DIAMOND v2.0.13 [30] blastx against the NCBI nr database (accessed on 24 March 2023) (–very-sensitive, e-value 10^−8^, −k 3).

### 2.4. Phylogenetic Analysis

Two phylogenetic trees were constructed using the assembled astrovirus contig: with polymerase fragment and with capsid protein. Protein sequences for corresponding ORFs were extracted from the contig using NCBI ORF finder and NCBI Blastx [31]. For the capsid protein, sequences and a full GenPept file were downloaded from the NCBI Protein database [32] using the query *txid39733[Organism:exp] capsid*, and for the polymerase gene using queries *txid39733[Organism:exp] orf1b* and *txid39733[Organism:exp] orf1ab*. From these files, sequences were selected for phylogenetic tree construction. A complete list of accession numbers is provided in Supplementary Materials S5 and S6. Sequences for the catalytic core domain of RdRP were extracted using coordinates from the GenPept file. Alignments were performed using MAFFT v7.490 [33] in E-INS-I mode. The trees were built in IQ-TREE v2.1.3 [34] with automatic model selection. Visualization was performed in iTOL [35].

### 2.5. Data Processing after SMART

The sequenced reads were processed using Trimmomatic v0.39 with the following options: ILLUMINACLIP: NexteraPE-PE.fa:2:30:10 and ILLUMINACLIP: adapters.fa:2:30:10 with LEADING:10, TRAILING:10, SLIDINGWINDOW:4:25, and MINLEN:40. The “adapters.fa” (Supplementary Material S2) file contains the 3′ MINT adapter and its reverse-complementary sequence. After inspecting reads, we found that not all adapter sequences were trimmed; therefore, Cutadapt v3.4 [36] with the non-internal adapter option was used for further trimming to cut off the adapters at the ends of the reads, followed by trimming two nucleotides from both ends of each read. Contigs were assembled with MEGAHIT v1.2.9 [29], and then in the assembled contigs, we endeavored to find the one that contained the original read, from which the primer was made. To do this, the resulting contigs were mapped to the read using minimap2 v2.23-r1111 [37].

Reads were mapped on contig 2 (described further in Section 3) using bowtie2 v2.4.4 (option –local) [26] and processed with samtools v1.15.1 [38].

## 3. Results

### 3.1. Metagenomic Sequencing of Bat Fecal Samples

In the present study, we performed metagenomic sequencing of 20 fecal samples from following bat species: Nyctalus noctula (12 samples from Saratov region and 3 from Rostov), Vespertilio murinus (1 sample from Voskresensk, 2 samples from Naro-Fominsk, and 1 sample from Moscow), and Pipistrellus kuhlii (1 sample from Astrakhan). Table S1 (Supplementary Materials) summarizes the statistics for the number of reads and contigs at different stages of the pipelines. The average percentage of reads removed by the host filtering was 17%. Reads were taxonomically classified using the Kraken2 program, and there were 32 estimated viral orders (*Algavirales*, *Amarillovirales*, *Articulavirales*, *Asfuvirales*, *Bunyavirales*, *Chitovirales*, *Cirlivirales*, *Cryppavirales*, *Durnavirales*, *Geplafuvirales*, *Ghabrivirales*, *Hepelivirales*, *Herpesvirales*, *Imitervirales*, *Jingchuvirales*, *Lefavirales*, *Martellivirales*, *Mononegavirales*, *Nidovirales*, *Norzivirales*, *Ourlivirales*, *Patatavirales*, *Piccovirales*, *Picornavirales*, *Pimascovirales*, *Reovirales*, *Rowavirales*, *Sepolyvirales*, *Stellavirales*, *Tubulavirales*, *Tymovirales*, and *Zurhausenvirales*).

The most represented order found in the samples was *Piccovirales* (the leftmost one in Figure 2). This is due to the high content of viruses from the subfamily *Densovirinae*, reaching up to 30% in some samples according to Kraken results. The number of insect viruses (*Genomoviridae* from the order *Geplafuvirales*, order *Lefavirales*) is also high in the samples. The second order on the heat map is *Picornavirales*, also represented mainly by insect viruses (families *Dicistroviridae*, *Iflaviridae*). A wide variety of mammalian viruses were also detected in the samples: adenoviruses, papillomaviruses, astroviruses, rotaviruses, and herpesviruses (their respective orders: *Rowavirales*, *Zurhausenvirales*, *Stellavirales*, *Reovirales*, and *Herpesviruses*). According to Diamond results in five samples (6, 11, 15, 17, and 4_23), interestingly, fragments of elephant endotheliotropic herpesvirus genome were found in bat feces, although this virus is known to infect only elephants.

A nearly full-length (6541 bp) astrovirus genome was assembled from sample 7_N. noctula_nextseq_Rostov (GenBank: OR552421; corresponding raw data: SRX21716486). Phylogenetic analysis (Supplementary Materials, Figures S1 and S2) of the capsid protein and the conserved catalytic core domain of RdRp showed their close similarity with other bat astroviruses. The nucleotide identity of this astrovirus with already known ones was less than 70%.

### 3.2. A Neural Network Approach for Finding Presumptive Viral Reads

It is interesting to note that many of the reads obtained (ranging from 3 to 34% across samples) are not identified with the Kraken2 program. To date, modern approaches to the search for new viruses based on deep learning methods (convolutional neural networks, transformers) have actively started to appear. We looked at one such program—DeepVirFinder [7]. This neural network was trained on viral and prokaryotic data. It consists of a convolutional layer, a max pooling layer, a dense layer with reluctance activation function, and a final dense layer with sigmoid function. To preliminarily evaluate the correctness of DeepVirFinder’s performance, the following study was conducted. Using the Diamond Taxonomy report, we selected 50 random contigs of each domain (eukaryotes, bacteria, and viruses) for the *16_N. noctula_nextseq_Saratov* sample and fed them into the input of the neural network. We hypothesized that, despite the possible presence of Diamond false positives, the overall trend of DeepVirFinder performance should correlate with the conditional a priori knowledge that a particular contig belongs to one of the domains.

**Figure 3:**
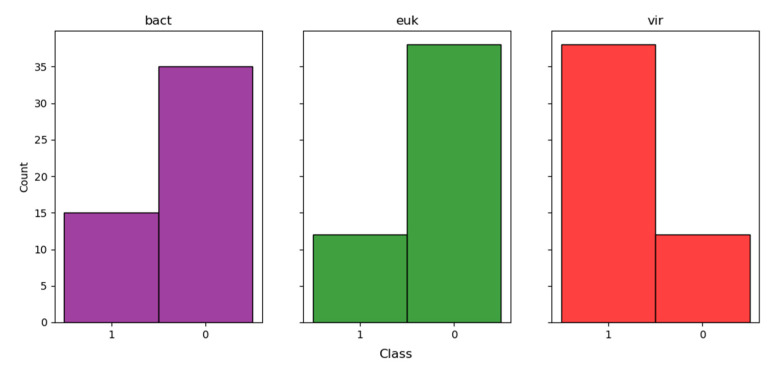
Results using analysis with DeepVirFinder for 50 bacterial, 50 eukaryotic, and 50 viral contigs identified using the Diamond program. The number of non-viral (class 0) and viral (class 1) contigs is shown for all three domains.

As can be observed in Figure 3, indeed, enrichment (37 out of 50) by viral contigs is observed only in the iral domain. However, it is worth noting that for bacteria and eukaryotes, we have also received a notable proportion (15 and 12 out of 50, respectively) of viral contigs. On the one hand, as previously mentioned, this may be a false positive Diamond result—some of the actual fragments of the viral genome may have been incorrectly classified as bacterial and eukaryotic. On the other hand, it may also be a false positive result of DeepVirFinder.

Once we were satisfied with the DeepVirFinder’s results, we assessed all the reads that Diamond could not classify with it.

**Figure 4:**
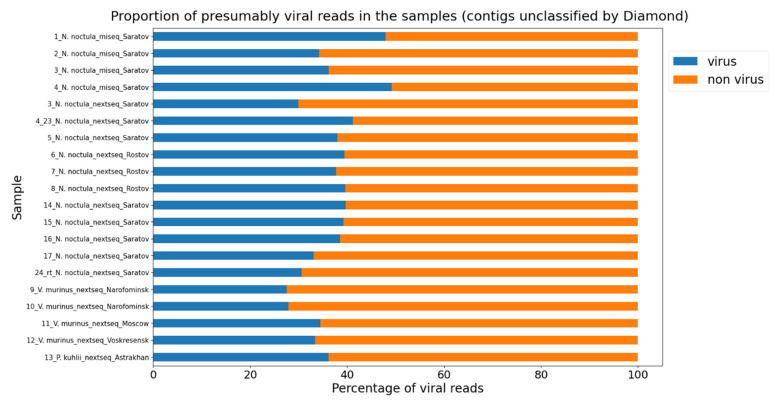
DeepVirFinder predictions for unclassified Diamond contigs for all samples in this study. The blue color indicates the presumably viral contigs.

As can be observed in Figure 4, approximately one-third of all reads for each sample may be viral in nature, which, based on the fairly high accuracy of the DeepVirFinder, indicates that a significant portion of the metagenomic data may contain hitherto unknown viruses.

### 3.3. Use of the SMART Method for Amplification of Target RNAs

**Figure 5:**
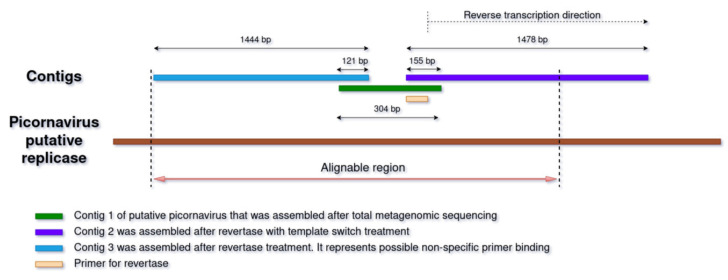
Mutual arrangement of contigs and primers for picornavirus revertase and replicase.

**Figure 6:**
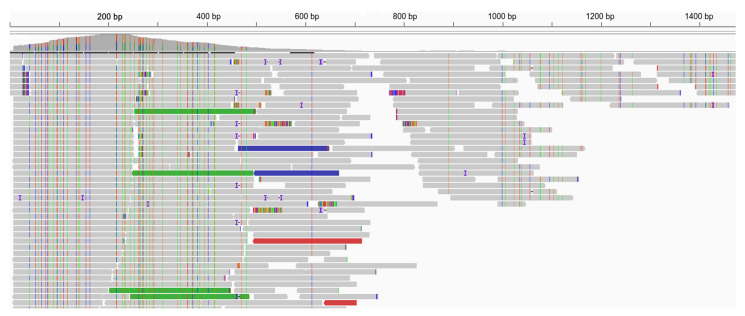
Mapping reads on assembled contig 2 after revertase treatment with template switching activity. Screenshot from the IGV browser.

With the above in mind, and in order to offer a possible solution for detecting novel viruses in metagenomic data, a specialized primer was designed as a case study for one read with extremely low homology, and the SMART method was used to obtain a longer genome fragment (see Section 2 for details). More specifically, the use of a revertase with template-switching activity to produce a longer viral fragment was tested on a read that exhibited very low homology with a known picornavirus (probably, *Jaksystermes virus*). After the samples were subjected to metagenomic sequencing as described above and contigs were assembled, one contig with a length 304 bp (hereafter referred to as “contig 1”), possessing less than 30% amino acid sequence homology with a known virus, was detected using BLASTX (shown in green in Figure 5). We then prepared a primer with an adapter ligated to it to perform reverse transcription using a revertase with “template switch” activity capable of attaching oligo-dCs without matrix. We then sequenced the SMART product of the sample containing contig 1 and re-assembled the contigs, among which we found two new contigs overlapping with contig 1 (Figure 5 and Figure 6).

Contig 2 (purple in Figure 5), which is the target product of the reaction and whose sequence begins with the primer, overlaps with contig 1 (green) for 155 bp, and has a final length of 1478 bp. Contig 3 (blue in Figure 5), which overlaps with contig 1 for 121 bp, has a length of 1444 bp and appears to be a result of nonspecific primer annealing. SMART-recovered contigs have very high similarity to the original contig in the overlapping regions. Contig 2 exhibits 99% similarity (154 bp out of 155 bp are identical), and contig 3 exhibits 100% similarity (121 bp out of 121 bp are identical). It should be noted that when reads are aligned, the depth of coverage on contig 2 is up to 9 times higher than on contig 3.

The main question is whether contig 2, which is essentially elongated using revertase version of contig 1, really belongs to a picornavirus. It is difficult to test with simple pairwise alignment because of the very low similarity of contig 2 to any known viruses. To test this in more detail, a consensus sequence (Supplementary Material S3, merged_contig) was constructed from contigs 1, 2, and 3, in which an open reading frame (Supplementary Material S4, ORF_from_merged_contig) whose amino acid sequence was searched against the Uniref_30 database using HHblits was selected [39]. The search results (E-value = 6.8 × 10^−67^) clearly indicate that this virus belongs to the *Picornaviridae* family and is most similar to *Jaksystermes virus*. Thus, the elongation compared to the initial contig was 1323 bp. It should be noted that the right end of contig 2 contains an ORF not detected with the BLAST and HHblits programs. However, since the left end of contig 2 most likely belongs to a picornavirus, the rest of contig 2 is also likely to be so, although it has no detectable homology with known pathogens.

Figure 6 shows the reads mapping to contig 2, assembled after the SMART procedure. The contig has a reverse-complementary orientation relative to the *Picornaviridae* sp. genome, so the revertase completes it from left to right. A sharp increase in the coverage from 200 at the beginning and up to 900 can be observed at this location, which then drops gradually, which is attributed to the use of Nextera XT kit for library preparation and the extent of RNA degradation. It is significantly higher than the depth of coverage by contig 3, which ranges from 10 to 100, demonstrating the high efficiency of amplification using the SMART technique.

The picornavirus, whose genome fragment was read by us, is most similar to *Spodoptera exigua virus AKJ-2014* or *Jaksystermes virus*, according to the ORF_from_merged_contig search on HHblits (Supplementary Material S4). Both of these viruses have been found in insects, which is in good agreement with the fact that bat feces contain large amounts of insect viruses.

## 4. Discussion

In this study, we performed metagenomic sequencing of bat fecal samples collected in different cities of the Russian Federation. The sequencing revealed a wide range of viruses, including mammalian, insect, and plant viruses, which is an expected result given the fact that bats themselves are a known reservoir of viral pathogens and that their diet often consists of insects [40]. A prime example is the numerous reads from the family *Densovirinae*, accounting for up to 30% of all sequencing reads in some samples, most of which probably belong to insect viruses. Other examples of viruses capable of infecting insects that we found in bat feces include the following: *King* and *Rold viruses* [41], *Cricket Iridovirus*, *Pidgeon bunyavirus*, *Icha Creek insect virus*, *Mothra virus*, and *Lampyris noctiluca tymovirus-like virus 1*. The mammalian viruses detected included adenoviruses, papillomaviruses, astroviruses, bastroviruses, rotaviruses, and herpesviruses. It is important to note that during the study, almost complete genomes of new bat astroviruses and bastroviruses were obtained, and the corresponding nucleotide sequences were deposited in genomic databases.

However, in this study, we aimed to validate the feasibility of using nucleic acid amplification methods from single primers, such as SMART [13], rather than to assess viral diversity. This is because a significant number of reads have no notable homology with previously known genomes of viruses or other organisms, and depending on the sample, from 3 to 34% of the reads did not show any detectable similarity to anything within the NCBI database, hinting that they might represent fragments of (yet undiscovered) viral genomes. As described above, metagenomic studies often produce a significant number of reads of DNA molecules that are difficult to identify because they have no homology with known organisms or viruses, and this information is best stored until something homologous becomes available in reference databases and/or more sensitive bioinformatics methods of genome identification are developed. In this regard, amplification methods from single primers may be useful as they provide extended regions of the genome that were not covered in the original study.

In the sequencing application, SMART technology was initially described as a fast, simple method for constructing full-length cDNA libraries for mRNA sequencing [42]. First-strand cDNA synthesis starts with a 3′-primer that includes an oligo(dT) sequence for annealing at the polyA+ site of the RNA. The SMART technology is based on the ability of MMLV revertase to add several non-template nucleotides, primarily dC, to the 3′-end of the growing first strand of cDNA, allowing annealing of an oligonucleotide with a known adapter sequence at the 5′-end. As a consequence, the revertase performs template switching and continues cDNA synthesis to the end of the oligonucleotide. The primer used to start reverse transcription (3′-primer) also contains an adaptor sequence at the 5′-end. Thus, the resulting (long) cDNA fragment is flanked by adapters with a predetermined nucleotide sequence, which allows its subsequent PCR enrichment. Thereby, the integration of oligo(dT) priming and SMART technology provides unbiased coverage of full-length mRNA regardless of the presence of rRNA or genomic DNA.

In this case, by applying a 3′-primer specific for a read that has low genetic similarity to known viruses, we can obtain a longer sequence of such a fragment, allowing for more accurate characterization and classification. For our example, we chose a read that has very low homology with a known picornavirus and applied the SMART method, which yielded a significantly longer fragment of the viral genome. Picornavirus was not chosen on the basis of its pathogenicity, but because it met the criteria for use of the SMART technique. It should be noted that in addition to the expected elongated region of the genome in the right 3′-5′ direction, we also obtained a fragment in the opposite direction, albeit with much lower coverage. The most likely explanation is nonspecific primer annealing, although this requires further confirmation.

Thus, we have demonstrated the potential of the SMART method for amplification and subsequent sequencing of extended segments of (novel) RNA virus genomes. This may allow the isolation of elongated fragments, which can potentially provide higher resolution as they can encompass relatively conserved genes such as, for example, RNA polymerase. However, one major drawback of the method is the usual high degree of fragmentation of RNA molecules in biological material, which inevitably limits the extent to which the read fragment of the genome can be elongated. For this reason, the method is applicable to biological material that was either immediately deep frozen upon receipt or promptly taken for experimentation. Nevertheless, this approach becomes particularly relevant in light of the development of machine learning-based approaches for predicting potential viral reads, which may facilitate the design of primer structures and further sequencing of extended stretches of DNA. For example, a bioinformatic tool for predicting fragments of viral genomes based on neural networks has shown that about one-third of all such unknown DNA reads may belong to viral pathogen genomes. Thus, the approach described in this paper can be used to detect novel viruses that have no significant homology with previously known viruses, although it requires additional experimental procedures after metagenomic sequencing. Nevertheless, as machine learning strategies for identifying reads in metagenomic sequencing data evolve, our proposed methodology may prove useful for the detection of novel viral pathogens.

## Data Availability

Data presented in this study are available upon request from the corresponding author. Scripts and results are available at https://github.com/lgilabs/SPAV, accessed on 31 August 2023. Assembled astrovirus genome from the sample 7_N. noctula_nextseq_Rostov was uploaded to GenBank (OR552421), and raw reads were uploaded to SRA (SRX21716486).

## Supplementary Materials

The following supporting information can be downloaded at: https://www.mdpi.com/article/10.3390/microorganisms11102532/s1. S1: A read containing a putative picornavirus; S2: adapters.fa; S3: merged_contig; S4: ORF_from_merged_contig; S5: Accession numbers for NCBI sequences used for astrovirus capsid phylogenetic tree; S6: Accession numbers for NCBI sequences used for astrovirus RdRP phylogenetic tree; Figure S1: Phylogenetic maximum likelihood tree of the astrovirus capsid gene; Figure S2: Phylogenetic maximum likelihood tree of the astrovirus RNA-dependent RNA polymerase gene; Table S1: The number of reads and contigs at different stages of the pipeline. Paired end reads were counted as single reads.
