# Supplementary material for: Unlocking the Viral Universe: Metagenomic Analysis of Bat Samples Using Next-Generation Sequencing
Source: Microorganisms. 2023 Oct 10;11(10):2532. doi: 10.3390/microorganisms11102532 (PMC10608967; doi:10.3390/microorganisms11102532)
Supplement: Supplementary file 1 [file microorganisms-11-02532-s001.zip › microorganisms-2620000-supplementary.pdf]

Supplementary materials

1. The read containing a putative picornavirus

AATCGTCCAAGAACAGTCTGCATGAGAGAGCGTTCCAACCCCAGCAATGAAGCAAGGGTTA
GAGCACTCCCAGCGATTTGCGAACCTGAGTCCGCACTCGCGAGGTTTGACAATGCAGCAGAA
ATCCCAATGGGACGCGCCTGCGCATTTGACAAAACGCTTTGAATGGTCGCTCCACCTGTGATA
GCGGAGGTACCAACATCACGTAATGCCGAACATGCCTTTTCAGACATGTCATCAATGGTTTTG
AGTGCGGT

2. adapters.fa

>1
AAGCAGTGGTATCAACGCAGAGTAC
>1_rc
GTACTCTGCGTTGATACCACTGCTT

3. merged_contig

GTCCGGGAGGTGGGGGAGGAAGGTCTTCGAGATCCGAATCAGAATCATCAGACCAAAGCTC
ACCCGAGCATTCTTCCAGAAGAGCATCAAGATCCTCATCGGAAGAATCGCTTTGAGCTTGAA
AATGCTGAAGTTGAGTTTGAAACTTATCTTCAGACATTTGCACGTTCTTGGCGATGGCGGCAA
CGATGTCTTCCAACTTGTGGAAGGCAGCGCAAGGATTACCGCCTTTGTTCCAGCCGCAGCCAG
TGGCAGAATTGCCAAAAGCACACGAACCAGACATTGAAGTCACGTGAAACTCCAAGTGTGA
AAACTCGGGGTCGTACGGATCTTCGGGAGCAGGCCTTGCCTTACCAGACTTCAAACGAGCCT
GAATAAGGAAAGGAAACCGACGATGTAACGCATCAACATTATTGATGGTAATGCTCTTGTGA
GGGAGACGATTACATGTTACAATCACCAGTCTGCTCTGGTAGGGCATCCCTTTCTGTGCGAGT
TGAGCTTGCACGGTACCAATACACTCACTCGAGATGAATGTAAGGTACATGGGATGATCCTT
GTTCTCCAGATCGGAGAACGCGTCATCAGCGTAAGTAATCTCTTGACCATTGTAGCCTTGATC
ATACTCATCACGCTGTTGAACGGACCAACGAGTCCATTTCTCTGCATCCTGAAAAGCAGTGTA
CTTTTGAGGGTCGTCAGACGCCAATTTCGACAACTTTTTGGTTACACGAGCTTGGATTTCGTTG
ATCAACCACGTTTTCCCAATTTGGGAGTCACCTTGAATGGCAACCCCAACTGGGCAGGGTCTG
AAGCCAGAAGTGGCTCTGACCCCCGTGATGATTTCATCCCAGGCCATGGCTTTCACCGAGAC
CGTGGAGATATTGACAAAAAGACTCGAATTCTTTAAGTCAGGACACGTGTTACGAGCACACG
TGTCAACAAGAGTTCTCAAGGAAGCGCGAAATTCTTCGTACTGCCGAGAGTATCTTGGAATG
AGGAACTTTGAGGGTTCGACCGCGATGATCTTTGCAATCTCCTCGGCGTCTTTAGACACCTTG
GTAAATGCATCTCGCAGTTCCGCAAGGTAGGCATACTTTCCCGTGGAAACTATACCTATGGAG
TTGAGGTACTCTTCGAGAGTATCCCAAACATCCTCCAGGGCTTTAGATTGATCCCTGGCTGTTT
TCAACCACTTCAATTGGTCGGAAACGACTGGGACTCCAAAGAGGCTCGAAAGCATTCCTCCA
AGTCCGAGAAGTCGTTTACGATCCCATGAGGTGGCCGTCATCCGGTCGAATCTTGCACGGAT
ATCGTCCAGACTTTGCGTCTTTGGGAGGCTTCCGAGGCCTCCCGCCGCCGCGTTTGCAAATCG
TCCAAGAACAGTCTGCATGAGAGAGCGTTCCAACCCCAGCAATGAAGCAAGGGTTAGAGCA
CTCCCAGCGATTTGCGAACCTGAGTCCGCACTCGCGAGGTTTGACAATGCAGCAGAAATCCC
AATGGGATGCGCCTGCGCATTTGACAAAACGCTTTGAATGGTCGCTCCACCTGTGATAGCGG

AGGTACCAACATCACGTAATGCCGAACATGCCTTTTCAGACATGTCATCAATGGTTTTGAGTG
CGGTTGTCCGCACTCCCTCAGTGGTGTCCTGAATCTGCTTGAACAGATGTTCCGTCCAAACAA
AAACAAAACGGTCGCCAAGGTAACCAAGGAACCCAAGTCCAGCTAGAAGGCCGGCTCCAG
GAACGTGTGCGAAGGACAAAAGAGTCCACACGATTGTCCAAAGACAAAGTGCGTAAGGCTT
ACGAACGTGCCTTACAAAATGAGCTGAAAATGAATCCAGCCCGGGACCCAAATCGGGCATG
GCAGACCACGCCGAGATGAGGAGACCTTTGGTTTGTTTAAGAACTGACATGGTTATTAAACG
GAAGGGGGTTGCACAATAGCAGTTGTGCTTCCTGTTGCTTCACTGGGAGAAGCGGGGAGAGG
AACCCAGAAGAATTCCTTGGGAAGGGACTCTTCCGACTCTGAATCAGAATCAGACAATTCCT
CAAGAAGGGAGAGAATGACATCAGCATGCGGATGCTTGCTAACACATTCTTCCCTAGGGGCG
GCTTCTACTGACGCGAGTTCAGTAAAACCAGGGAGGGGAAGAGAAATTACATCAGGATGTA
CGTCACCGATGCAATCCTCAGGTGGGGGAGGAATAAGATCCCAAATTTGAAGATCGGGATCT
AATACTCCAATCTCCGGGTAGGGAGGGAGTACCTCACCATCAGTCGAGAGGGGCCCAGTGG
AACCCTTTATGGGGGAAAGCCACTTGACCTTCTTGACTGCAGCCGTTGAAGTCTTGACGGATT
CAACAACGACTTTGCTAATCTTGGCAGGAAAAACCTCCTTGCCTTGAGAAGCATCCCAAGCA
GAAAGCTTCGATGGGCGCGCAAGACGCTCAATCTTAGCTCCCTCTGGAGCTTTGGGCACGTAT
GGGACATACATGTATCCAAAGAATTGGGCAGTGGTATTACTACCATACCCATCCAAAAACGA
ATGACGCCAACCTTCAAAGGTGGCGGCACCAGTCATGGAAACCGGAGTTGAAGTCACAGGG
ACAAACAACTTCGAGAATTTGGAAACAGCTTTGGCTTTCGCCGGAAGCTTCTCCAAAAGAGC
GTTAAGAGCCTTGTTACGGGCCTCAACCTCTTGCGTTCGAGCAATGCGTTGATTTCTAGTCGTG
CATTCCTTGAACGGAGTGACAGGGAAGTCTTTGTAGACTTTCCTCTTCACAGTCCAAGAAAGC
TTGGAAAGAGCTTCCTCAGACGGAATGGGAACACGCGTTTCCTTGGGTTTAGCAGGCTGCGA
AACAGTCTGCTTACCGTCAGAAACGGCGTTCTTTTTGGAGACACCAGCAAATGTCTCCTCACG
CCGCAAAGCCTTATTACAGA

4. ORF_from_merged_contig

MSVLKQTKGLLISAWSAMPDLGPGLDSFSAHFVRHVRKPYALCLWTIVWT
LLSFAHVPGAGLLAGLGFLGYLGDRFVFVWTEHLFKQIQDTTEGVRTTAL
KTIDDMSEKACSALRDVGTSAITGGATIQSVLSNAQAHPIGISAALSNLA
SADSGSQIAGSALTLASLLGLERSLMQTVLGRFANAAAGGLGSLPKTQSL
DDIRARFDRMTATSWDRKRLLGLGGMLSSLFGVPVVSDQLKWLKTARDQS
KALEDVWDTLEEYLNSIGIVSTGKYAYLAELRDAFTKVSKDAEEIAKIIA
VEPSKFLIPRYSRQYEEFRASLRTLVDTCARNTCPDLKNSSLFVNISTVS
VKAMAWDEIITGVRATSGFRPCPVGVAIQGDSQIGKTWLINEIQARVTKK
LSKLASDDPQKYTAFQDAEKWTRWSVQQRDEYDQGYNGQEITYADDAFSD
LENKDHPMYLTFISSECIGTVQAQLAQKGMPYQSRLVIVTCNRLPHKSIT
INNVDALHRRFPFLIQARLKSGKARPAPEDPYDPEFSHLEFHVTSMSGSC
AFGNSATGCGWNKGGNPCAAFHKLEDIVAAIAKNVQMSEDKFQTQLQHFQ
AQSDSSDEDLDALLEECSGELWSDDSDSDLEDLPPPPG

5. Accession numbers for NCBI sequences used for astrovirus capsid phylogenetic tree

WBM84735.1, WBM84741.1, ACN88708.1, WBV74327.1, UQK62207.1, ADJ17721.1, BDS38213.1, QCC21360.1, UYR25368.1, WEP24395.1, UQR79056.1, UYB78877.1, AFK92935.1, UPO37649.1,

AFK92943.1, AFK92952.1, QOQ34712.1, WAK45937.1, BAX00245.1, BAX00266.1, UKG18743.1, AVX29485.1, YP_009345904.1, AVX29501.1, AEM37631.1, AEM37625.1, AEM37637.1, AEM37616.1, APA19827.1, APA19818.1, APA19845.1, APA19833.1

6. Accession numbers for NCBI sequences used for astrovirus RdRP phylogenetic tree

WDD45104.1, WDD45101.1, WDD45099.1, WDD45095.1, WDD45092.1, QOR29562.1, QFU14627.1, QBQ70678.1, ADJ38393.1, ADJ38390.1, ADJ38387.1, QCT05778.1, WIM52625.1, WIF19740.1, WIF19737.1, WFG33662.1, WFG33659.1, WFG33657.1, WFG33649.1, WFG33636.1, WFG33633.1, UVF58764.1, UVF58761.1, UZV41890.1, UZV41886.1, UZV41882.1, UZV41877.1, UJI09362.1, UIB21022.1, QNP14649.1, QNP14646.1, QOY58057.1, QWT72248.1, QWN59207.1, AWV67085.1, QBY26588.1, QBJ04611.1, YP_006905856.1, APQ41659.1, APQ41656.1, QJX57343.1, BDG57751.1, BDG57748.1, NP_751905.1, QZX51944.1, QRD80926.1, WBV73435.1, WBV73432.1, UVW93787.1, UVW93785.1, QSV51811.1, QSV51808.1, QDY92123.1, AWF70742.1, QBL55998.1, QDY92212.1, QDY92196.1, QDY92170.1, AYM47266.1, AEM37630.1, AEM37627.1, ACN88707.1, ACN78557.1, AGK45543.1, AEX15920.1, CAB95006.3, AFT63041.1, AFT63037.1, ABX46589.1, ABX46586.1, UWK09061.1, UUB69046.1, UUB69043.1, USC30037.1, QNJ35017.1, WCI13789.1

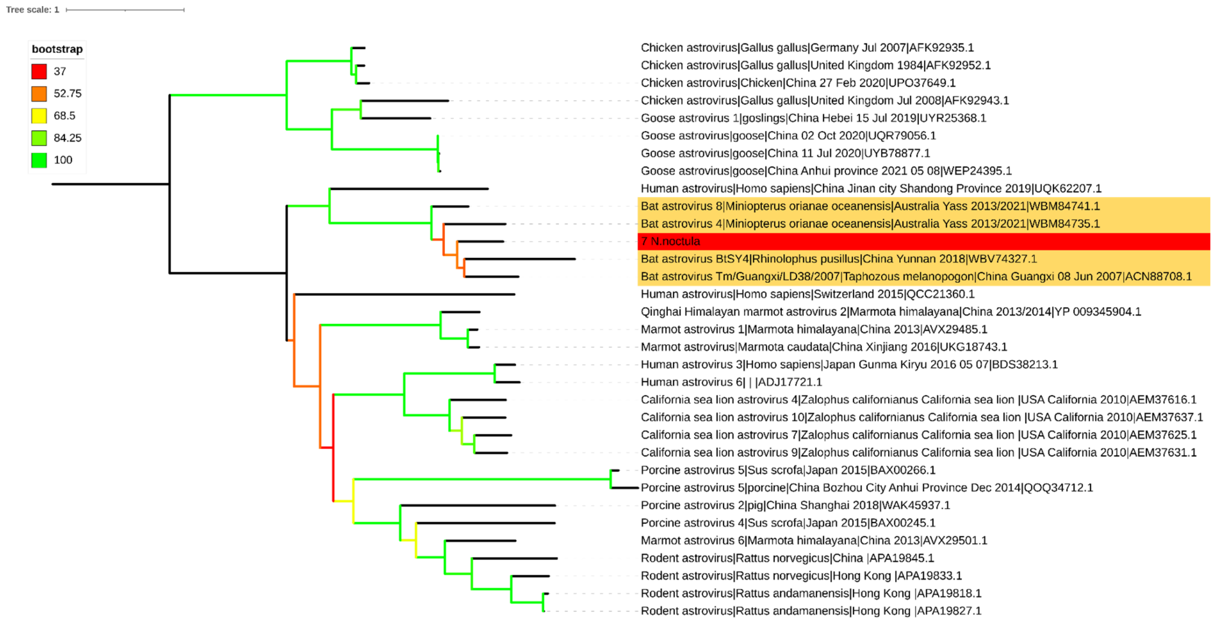

Figure S1. Phylogenetic maximum likelihood tree of the astrovirus capsid gene. The sample of this study is in red, the bat samples are in orange. The tree is rooted using the midpoint method.

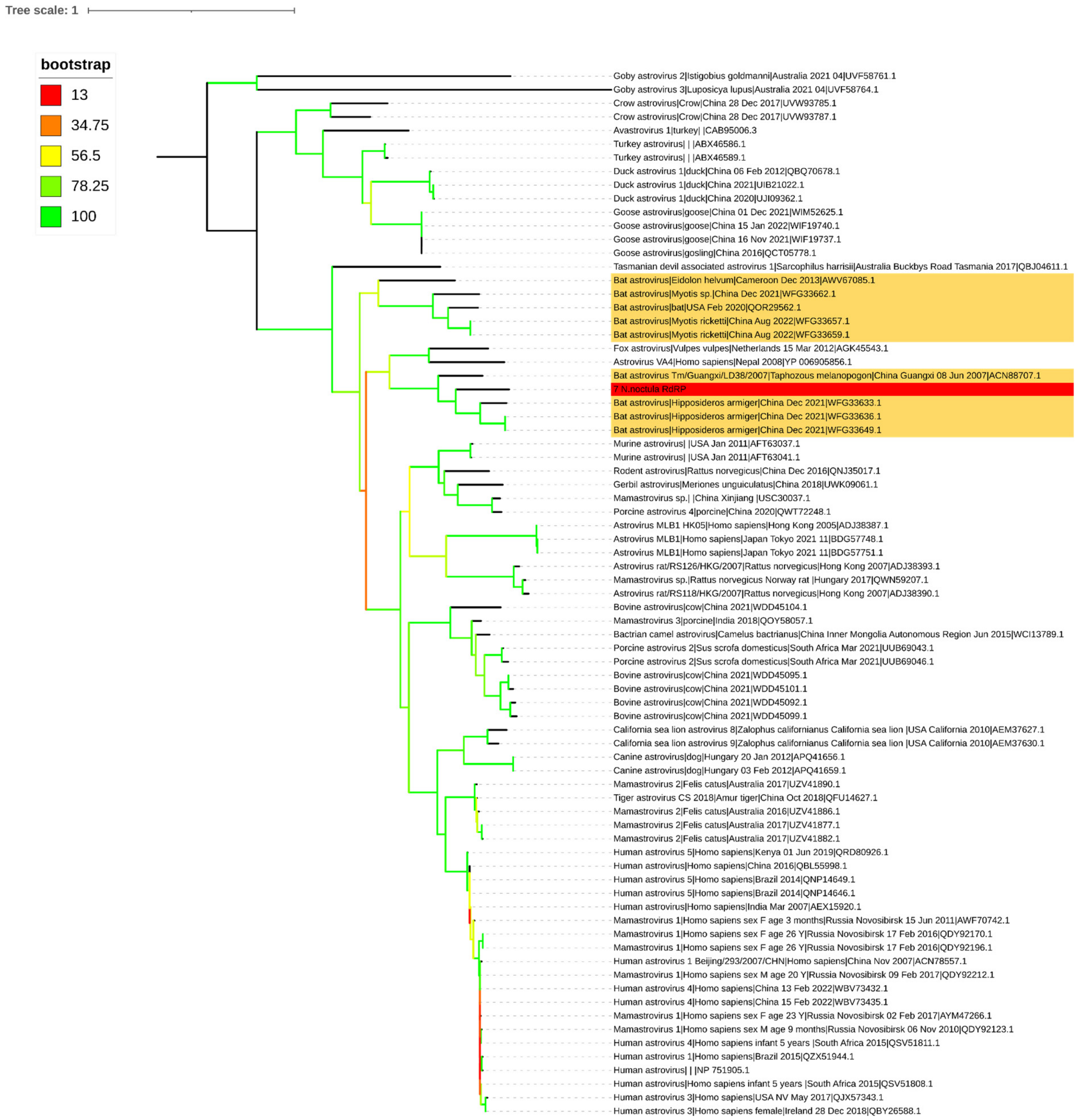

Figure S2. Phylogenetic maximum likelihood tree of the astrovirus RNA-dependent RNA polymerase gene. The sample of this study is in red, the bat samples are in orange. The tree is rooted using the midpoint method.

Table S1. The number of reads and contigs at different stages of the pipeline. Paired end reads were counted as single reads.

Sample	After paired reads merging	After host filtration with bowtie2	Percentage of filtered host reads	After filtration with Kaiju	Contigs
1_N. noctula_miseq_Saratov	1869552	1649224	11.8%	926225	7303
2_N. noctula_miseq_Saratov	2655726	2401644	9.6%	266056	6684
3_N. noctula_miseq_Saratov	3056611	2984903	2.3%	1194518	5917
4_N. noctula_miseq_Saratov	3257932	3117999	4.3%	1932943	6089
3_N. noctula_nextseq_Saratov	33495721	32451127	3.1%	5794122	9440
4_23_N. noctula_nextseq_Saratov	26681762	25956165	2.7%	5233477	14924
5_N. noctula_nextseq_Saratov	26944423	23004659	14.6%	11937717	31115
6_N. noctula_nextseq_Rostov	33697208	21657416	35.7%	7989802	89113
7_N. noctula_nextseq_Rostov	35039719	28211066	19.5%	6694664	20727
8_N. noctula_nextseq_Rostov	36080769	24049695	33.3%	11872927	88941
14_N. noctula_nextseq_Saratov	36049090	21532957	40.3%	6966677	50439
15_N. noctula_nextseq_Saratov	25289088	19534552	22.8%	3513424	22234
16_N. noctula_nextseq_Saratov	40138841	11290357	71.9%	9810470	229115
17_N. noctula_nextseq_Saratov	31736804	27644447	12.9%	8616341	22666
24_rt_N. noctula_nextseq_Saratov	28991264	28831690	0.6%	11327681	44478
9_V. murinus_nextseq_Narofominsk	23393606	22837965	2.4%	15187215	13828

10_V. murinus_nextseq_Narofominsk	32805451	32203270	1.8%	12913528	54453
11_V. murinus_nextseq_Moscow	27195614	23231340	14.6%	13913106	24379
12_V. murinus_nextseq_Voskresensk	17158740	13237388	22.9%	9481311	42476
13_P. kuhlii_nextseq_Astrakhan	8166109	7041760	13.8%	3742475	1805
